# A single centre prospective cohort study addressing the effect of a rule-in/rule-out troponin algorithm on routine clinical practice

**DOI:** 10.1177/2048872617746850

**Published:** 2017-12-04

**Authors:** Jack Marjot, Thomas E Kaier, Katherine Henderson, Laura Hunter, Michael S Marber, Divaka Perera

**Affiliations:** 1 King’s College London BHF Centre, St Thomas’ Hospital, London, UK; 2 Emergency Department, St Thomas’ Hospital London, UK

**Keywords:** High-sensitivity cardiac troponin T, acute coronary syndrome, rule-in/rule-out algorithm

## Abstract

**Aims:**

In 2015, the European Society of Cardiology introduced new guidelines for the diagnosis of acute coronary syndromes in patients presenting without persistent ST-segment elevation. These guidelines included the use of high-sensitivity troponin assays for ‘rule-in’ and ‘rule-out’ of acute myocardial injury at presentation (using a ‘0 hour’ blood test). Whilst these algorithms have been extensively validated in prospective diagnostic studies, the outcome of their implementation in routine clinical practice has not been described. The present study describes the change in the patient journey resulting from implementation of such an algorithm in a busy innercity Emergency Department.

**Methods and results:**

Data were prospectively collected from electronic records at a large Central London hospital over seven months spanning the periods before, during and after the introduction of a new high-sensitivity troponin rapid diagnostic algorithm modelled on the European Society of Cardiology guideline. Over 213 days, 4644 patients had high-sensitivity troponin T measured in the Emergency Department. Of these patients, 40.4% could be ‘ruled-out’ based on the high-sensitivity troponin T concentration at presentation, whilst 7.6% could be ‘ruled-in’. Adoption of the algorithm into clinical practice was associated with a 37.5% increase of repeat high-sensitivity troponin T measurements within 1.5 h for those patients classified as ‘intermediate risk’ on presentation.

**Conclusions:**

Introduction of a 0 hour ‘rule-in’ and ‘rule-out’ algorithm in routine clinical practice enables rapid triage of 48% of patients, and is associated with more rapid repeat testing in intermediate risk patients.

## Introduction

Chest pain and related complaints are estimated to account for 6% of all attendances to UK Emergency Departments (EDs).^1^ Determining which of these presentations represent an acute coronary syndrome, quickly and with high sensitivity and specificity, is an everyday challenge. The measurement of cardiac-specific biomarkers released into the circulation is invaluable, and the measurement of cardiac troponin (cTn) I and T is engrained in the universal definition of myocardial infarction.^2^ However, the slow release of cTn, in combination with the relative analytic insensitivity of conventional cTn assays, has necessitated serial measurements separated by at least six hours to increase both sensitivity and specificity. This period of diagnostic uncertainty prolongs the patient’s hospital stay, delays their treatment and has an associated fiscal cost. The advent of high sensitivity troponin assays has encouraged investigators to examine shorter intervals between repeat troponin estimations. The high sensitivity assays have also allowed the testing of diagnostic cut-off concentrations well below the population defined 99^th^ centile to rapidly rule out acute myocardial injury. These innovations culminated in the European Society of Cardiology (ESC) releasing new guidelines in September 2015 for the management of patients without persistent ST elevation.^3–6^ These guidelines adopt a ‘rule-out’ troponin value significantly below the 99^th^ centile and a ‘rule-in’ value well above the 99^th^ centile. Between these values of diagnostic clarity, the change in troponin level over the course of one hour can guide further rule-in or rule-out. In October 2015 we proposed introduction of the 0 hour rule-in/rule-out component of the ESC algorithm at St Thomas’ Hospital (based in central London and home to a tertiary cardiac unit) and adopted the guideline, following an internal consultation process, during December 2015–January 2016. This internal consultation process also involved extension of teaching to ED staff, both nursing and physician, as to the appropriate use of the algorithm. All ‘post-intervention’ data were collected after implementation and associated staff training.

Whilst the ESC guidelines help to streamline the diagnostic pathway, there has been little information regarding their impact on front-line medical services. The present study, based in the ED of a large Central London hospital, aims to (a) prospectively assess the risk classification of patients based on 0 hour hs-cTnT measurement, and (b) examine the effect of clinical implementation of the 0 hour component of the ESC guideline on the patient pathway. In particular, we document changes in the pattern of repeat troponin measurements and overnight admission.

## Methods

Data was prospectively collected on all high-sensitivity cardiac troponin T (hs-cTnT) assays performed on serum from patients presenting to the ED of St Thomas’ Hospital, between September 2015–March 2016. This time-period of data collection spans the pre-intervention (September-November), transition (December), and post-intervention (January-March) phases of algorithm implementation. hs-cTnT assays were performed using the Roche Elecsys platform (using a high-sensitivity reagent instead of a contemporary: 99th percentile of a healthy reference population reported at 14 ng/l, imprecision corresponding to 10% coefficient of variation (CV) at 13 ng/l, limit of blank at 3 ng/l, limit of detection at 5 ng/l). The hs-cTnT value measured in the ED was matched to any subsequent hs-cTnT measurement on the same patient within 24 h. Further information on admission, admitting specialty, and length of stay was collected from electronic discharge records. Data on presenting symptom was obtained from the system used for triage and clinical tracking in the ED (Ascribe Symphony); this captures the prime medical complaint but, however, it does not encompass a physician’s interpretation. Discharge diagnoses are locally recorded according to the 10^th^ revision of the International Statistical Classification of Diseases and Related Health Problems (ICD-10) and were subsequently categorised into diagnostic groups by two adjudicators (JM and TEK).

**Figure 1.**
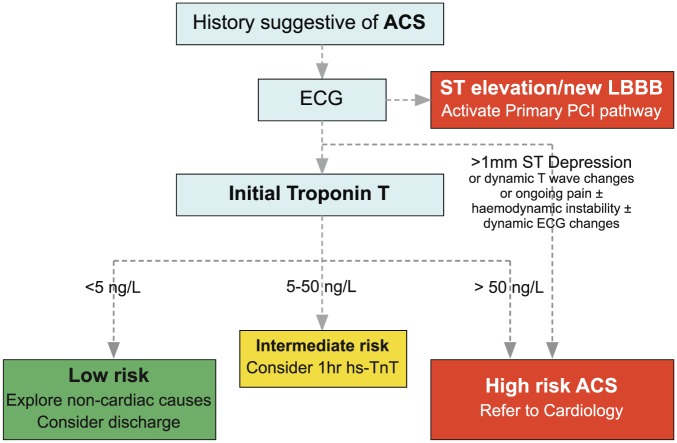
The high-sensitivity troponin T (hs-cTnT) rapid diagnostic algorithm introduced at St Thomas’ Hospital. ACS: Acute Coronary Syndrome; LBBB: Left Bundle Branch Block; ECG: electrocardiogram; PCI: Percutaneous Coronary Intervention.

The new algorithm for the diagnostic management of possible Non-ST elevation Acute Coronary Syndrome (NSTE-ACS) can be summarised as follows: hs-cTnT is measured on arrival to ED for patients with a history suggestive of Acute Coronary Syndrome (ACS), and an electrocardiogram (ECG) without persistent ST elevation. ACS can be ‘ruled-out’ in low-risk patients with a hs-cTnT on presentation of <5 ng/l, and ‘ruled-in’ for those patients with an initial hs-cTnT of >50 ng/l (Figure 1). Although not adopted into our algorithm, the ESC advises that in patients with an initial hs-cTnT of 5-51 ng/l, a repeat hs-cTnT at one hour is performed, with rule-out if the initial hs-cTnT is <12 ng/l and if a change in hs-cTnT (ΔTnT) is <3 ng/l, and rule-in if ΔTnT is ≥5 ng/l. For the purposes of our analysis, a patient was considered to have had a repeat hs-cTnT if a second sample was measured within 24 h of the first. Patients were excluded from analysis if the first sample haemolysed. Those hs-cTnT measurements returned below the limit of blank (<3 ng/l) were all ascribed a value of 2.99 ng/l to allow for data analysis. Continuous variables were assessed for normality using Shapiro-Wilk Test. All data are expressed as medians (1st quartile, 3rd quartile) or means (standard deviation) for continuous variables (compared with the Mann-Whitney-U test or student’s *t*-test), and for categorical variables as numbers and percentages (compared with Pearson chi-square). Hypothesis testing was two-tailed, and *p* values <0.05 were considered statistically significant. Statistical analysis was conducted using SPSS version 22 (IBM Corp., Armonk, New York, USA) and R, version 3.3.0 GUI 1.68 (The R Foundation for Statistical Computing), including ggplot2.

## Results

**Figure 2.**
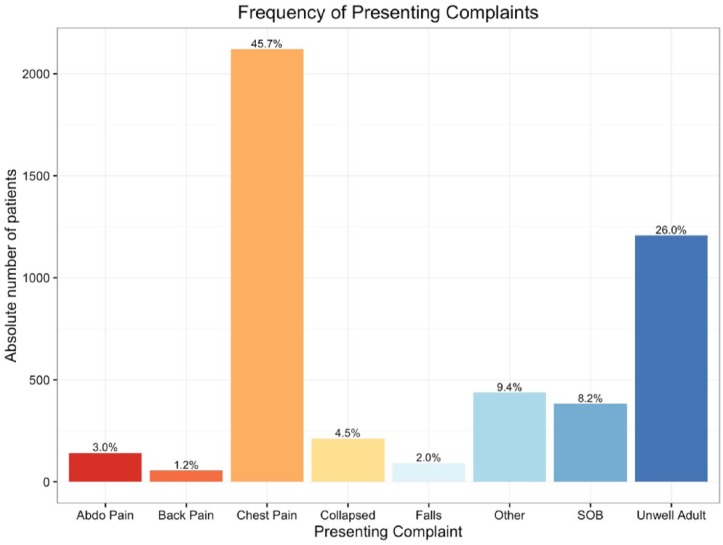
Bar graph summarising the presenting complaint of all patients (*n*=4644) with a measured high-sensitivity troponin T (hs-cTnT) in the entire study period; frequencies quoted as percentage of the cohort. SOB: Shortness of Breath.

Over a period of 213 days, spanning the introduction of the new diagnostic protocol, a total of 4644 patients had a hs-cTnT measurement in the ED. A summary of the presenting complaint of all patients with hs-cTnT measurements in the study period (September 2015 –March 2016) is presented in Table 1. In short, of the patients with a measured hs-cTnT (*n*=4644), chest pain was the primary presenting symptom in 45.7% (*n*=2120), and shortness of breath in 8.2% (*n*=382) – see Figure 2. Median age was 54 years (interquartile range (IQR), 41–70).

**Table 1.** Summary of the presenting complaint of all patients with high-sensitivity troponin T (hs-cTnT) measurements in the study period (September 2015–March 2016).

Presenting complaint	All attendances	(%)
**Abdominal pain**	141	(3.0)
**Back pain**	55	(1.2)
**Chest pain**	2120	(45.7)
**Collapsed adult**	211	(4.5)
**Falls**	91	(2.0)
**Shortness of breath**	382	(8.2)
**Other**	437	(9.4)
**Unwell adult**	1207	(26.0)
**Total**	*n*=4644	

Frequencies quoted as number (%); sample selection: all patients presenting to the Emergency Department with a hs-cTnT measured as part of their assessment between September 2015 and March 2016, age ≥18 years; ‘Other’ summarises non-cardiac presentations such as ‘overdose’ and ‘limb problems’.

### 0 hour risk stratification for whole sample period

**Figure 3.**
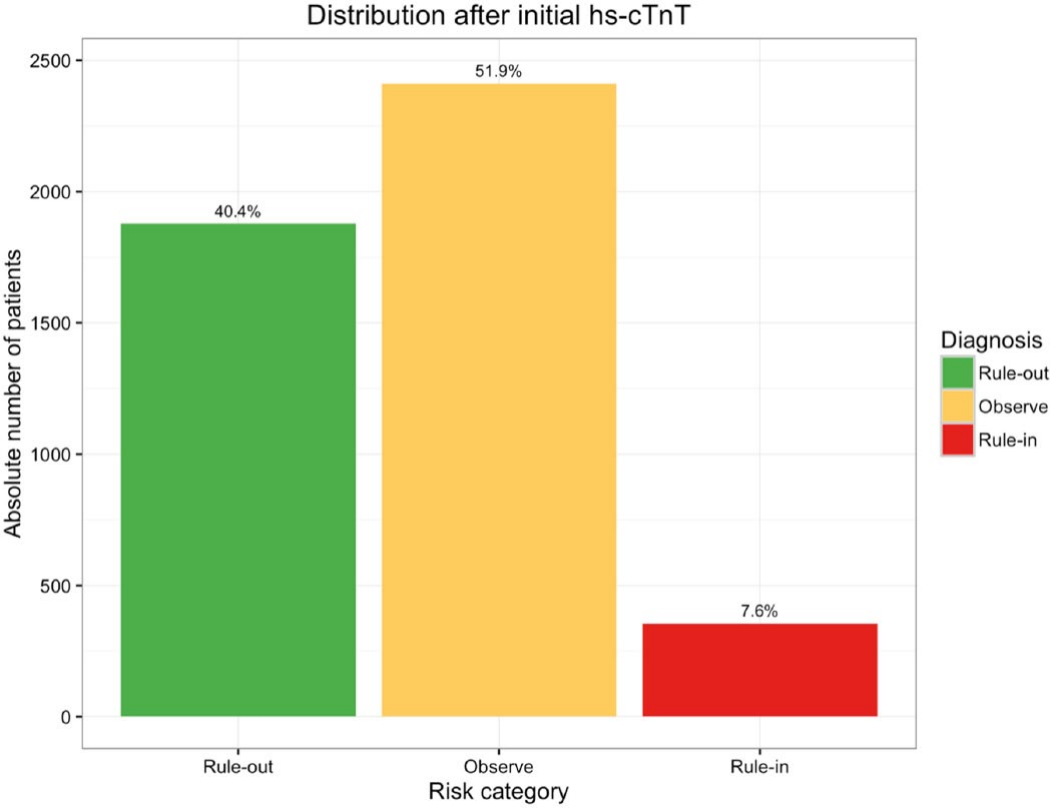
Graph outlining the distribution of all high-sensitivity troponin T (hs-cTnT) values measured on patients presenting to the Emergency Department during the monitoring period (September 2015–March 2016; *n*=4644); the following thresholds applied: <5 ng/l ‘rule-out’, 5-50 ng/l ‘observe’, >50 ng/l ‘rule-in’.

Of the entire cohort, 40.4% had an initial hs-cTnT concentration below the ‘rule-out’ value of 5 ng/l at presentation, and 7.6% had a concentration above the ‘rule-in’ value of 50 ng/l (Figure 3). Of the patients presenting with chest pain (*n*=2120), 1026 (48.4%) had an initial hs-cTnT concentration below the ‘rule-out’ threshold, 107 (5%) had a concentration above the ‘rule-in’ threshold. Of the patients presenting with shortness of breath (*n*=382), 89 (23.3%) had an initial hs-cTnT concentration below the ‘rule-out’ threshold, 74 (19.4%) had a concentration above the ‘rule-in’ threshold.

### Retrospective analysis of deltas for all presentations

Although our algorithm incorporates only the rule-in/rule-out classification based on a 0 hour hs-cTnT measurement, retrospective analysis of the entire cohort demonstrates that 10.6% of those at intermediate risk (0 hour hs-cTnT 5–50 ng/l) could have been ruled-in on repeat testing with a ΔTnT ≥5 ng/l, and 45.1% could have been ruled-out on the basis of an initial TnT<12ng/l and ΔTnT<3 ng/l.

### Discharge diagnosis

**Figure 4.**
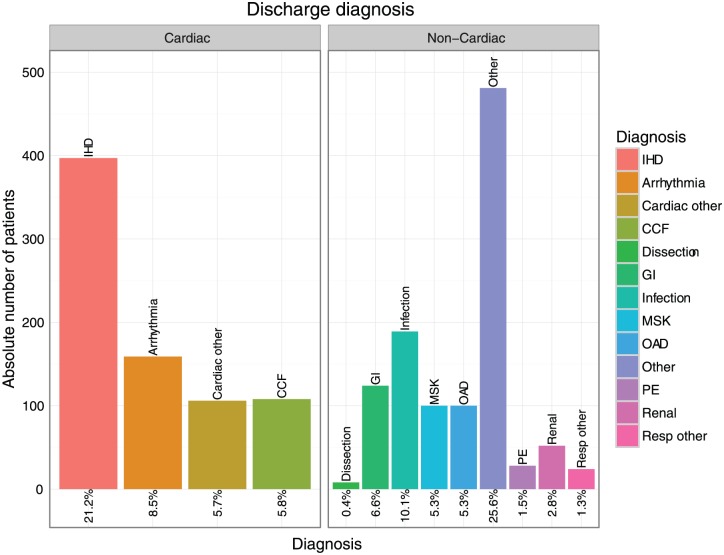
Bar graph summarising the discharge diagnosis of all admitted patients in the monitoring period (September 2015–March 2016; *n*=1876); frequencies quoted as percentage of the overall number of patients admitted following high-sensitivity troponin T (hs-cTnT) testing. CCF: congestive cardiac failure; GI: gastrointestinal disorder; IHD: ischaemic heart disease; MSK: musculo-skeletal disorder; OAD: obstructive airways disease; PE: pulmonary embolism. ‘Cardiac other’ includes myocarditis, valvular heart, conduction tissue and pericardial disease; ‘Resp other’ includes pleural effusion; GI includes gastro-oesophageal reflux disease, gastroenteritis and symptomatic cholelithiasis; ‘Infection’ includes lobar pneumonia, urinary tract infection and influenza; MSK includes costochondritis, bony fractures and other injuries; ‘Other’ includes sickle-cell anaemia, malignancy and mental health disorder.

**Figure 5.**
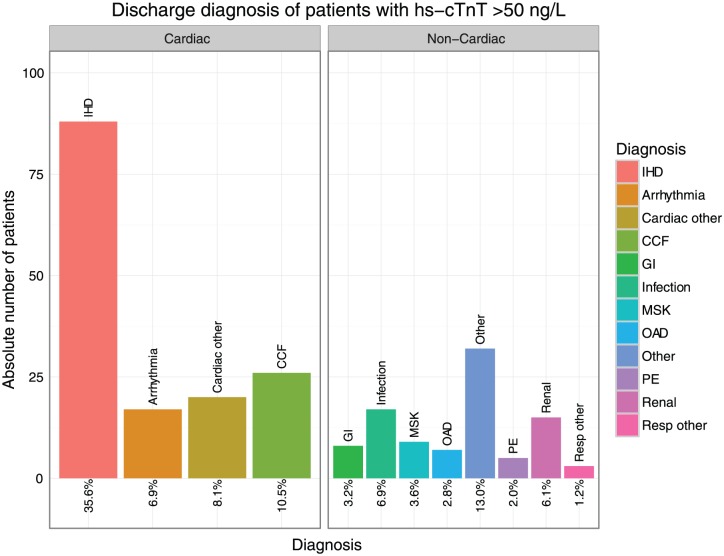
Bar graphs summarising the discharge diagnosis of all admitted patients in the monitoring month with an initial high-sensitivity troponin T (hs-cTnT) level >50 ng/l (*n*=247); frequencies quoted as percentage. CCF: congestive cardiac failure; GI: gastrointestinal disorder; IHD: ischaemic heart disease; MSK: musculo-skeletal disorder; OAD: obstructive airways disease; PE: pulmonary embolism. ‘Cardiac other’ includes myocarditis, valvular heart, conduction tissue and pericardial disease; ‘Resp other’ includes pleural effusion; GI includes gastro-oesophageal reflux disease, gastroenteritis and symptomatic cholelithiasis; ‘Infection’ includes lobar pneumonia, urinary tract infection and influenza; MSK includes costochondritis, bony fractures and other injuries; ‘Other’ includes sickle-cell anaemia, malignancy and mental health disorder.

Altogether 1876 patients were admitted from the ED during the entire study period. Amongst these, the prevalence of ischaemic heart disease in the discharge diagnosis was 21.2% (*n*=397); congestive cardiac failure was the discharge diagnosis in 5.8%; pulmonary embolism in 1.5%. Of those patients admitted with a troponin value above the rule-in threshold (50 ng/l), 35.6% were diagnosed with ischaemic cardiac pathology (see Table 2, Figure 4 for details on all admitted patients, Figure 5 for subgroup analysis on all patients with a hs-cTnT at presentation >50 ng/l).

**Table 2.** Details of admitted patients.

Coding diagnosis	All admitted patients	hs-cTnT >50 ng/L
**Aortic dissection**	8 (0.4)	0 (0)
**IHD**	397 (21.2)	88 (35.6)
**Arrhythmia**	159 (8.5)	17 (6.9)
**CCF**	108 (5.8)	26 (10.5)
**Cardiac other**	106 (5.7)	20 (8.1)
**PE**	28 (1.5)	5 (2.0)
**OAD**	100 (5.3)	7 (2.8)
**Resp other**	24 (1.3)	3 (1.2)
**Infectious**	189 (10.1)	17 (6.9)
**Renal**	52 (2.8)	15 (6.1)
**GI**	124 (6.6)	8 (3.2)
**MSK**	100 (5.3)	9 (3.6)
**Other**	481 (25.6)	32 (13.0)
**Total**	*n*=1876	*n*=247

CCF: congestive cardiac failure; GI: gastrointestinal disorders; IHD: ischaemic heart disease; MSK: musculo-skeletal disorders; OAD: obstructive airways disease PE: pulmonary embolism.

‘Cardiac other’ includes myocarditis, valvular heart and pericardial disease; ‘Resp other’ includes pleural effusion; Infectious includes lobar pneumonia, urinary tract infection and influenza; GI includes gastro-oesophageal reflux disease, gastroenteritis and symptomatic cholelithiasis; MSK includes costochondritis, bony fractures and other injuries; ‘Other’ includes sickle-cell anaemia, malignancy and mental health disorder. Frequencies quoted as number (%); Sample representative of the entire study period (September 2015 – March 2016) and comprises of all patients admitted from the Emergency Department.

### Repeat troponin samples in the post-intervention period

In the three months following introduction of the algorithm (i.e. the ‘post-intervention period’), 946 patients (50.2%) had an initial hs-cTnT in the 5-50 ng/l zone of diagnostic uncertainty – of these, 443 (46.8%) had a repeat measurement within 24 h. Of the patients undergoing further testing, 189 (42.7%) had a repeat measurement within 1.5 h. Median time to repeat hs-cTnT measurement was 1.6 h (1.3, 2.2) for the entire post-intervention period.

Eight hundred and ninety-two patients presented with chest pain in the post-intervention period. Of these, 390 patients (43.7%) were in the observational group, of which 222 (56.9%) had a repeat measurement within 24 h. Of the patients undergoing further testing, 106 (47.7%) had a repeat measurement within 1.5 h. The median time to repeat hs-cTnT measurement in the group presenting with chest pain was 1.5 h (1.3, 2).

One hundred and fifty-four patients presented with shortness of breath in the post-intervention period. Of these, 87 patients (56.5%) were in the observational group, of which 29 (33.3%) had a repeat hs-cTnT within 24 h. Of the patients undergoing further testing, 10 (34.5%) had a repeat measurement within 1.5 h. Median time to repeat in the group presenting with shortness of breath was 1.8 h (1.4, 2.1).

### Comparison of pre- and post-intervention periods for all presentations

Over the timeframe of implementation of the new algorithm we have demonstrated a gradual rise in the proportion of patients in the intermediate risk group (all presenting complaints) who had a repeat hs-cTnT measured within 1.5 h. At month 1 (pre-implementation), only 3.3% of repeat hs-cTnT measurements in the intermediate-risk patients were within 1.5 h, rising to 40.8% by month 7 (post-implementation) (*p*<0.001). In tandem, the median time to repeat troponin has fallen from 7.8 h (4.7, 11.1) to 1.7 h (1.3, 2.4) (*p*<0.001). This has been accompanied by a non-significant trend towards reduced overnight admissions in the low-risk group. In a month prior to implementation, of all patients with a hs-cTnT measurement <5 ng/l on presentation to ED, 12.7% were admitted for at least one night. This figure fell to 9.5% by month 7 (*p*=0.26, *n*=525). Early outcome data demonstrates that 30-day mortality in all patients with suspected ACS was not different before and after implementation of the new algorithm (1.8% versus 1.4% respectively, *p*=0.38).

## Discussion

This study documents the rate of adoption of a rapid rule-in/rule-out algorithm for the routine clinical care of patients presenting with suspected NSTE-ACS, based on a single blood test at presentation. In this large cohort of over 4600 patients, 48% of all patients and 53% of patients with chest pain could be dichotomised into high- or low risk groups on the basis of a single hs-cTnT measured on presentation.

Multiple studies have prospectively validated the sensitivity and specificity of diagnostic algorithms based on high-sensitivity troponin assays.^4–8^ The unifying aim is to rapidly identify patients with ACS, facilitating prompt therapeutic intervention for those who need it, and prompt discharge for those who do not. However, since the ESC guidelines have been established, there is a dearth of studies that have addressed the fundamental question – can such an algorithm be implemented into routine clinical practice? As we have incorporated our algorithm into clinical practice, we have seen an increased rate of repeat testing, and a trend to faster repeats, in patients classified into the intermediate risk group on presentation. Whilst this algorithm should lead to empowerment of clinicians to exclude NSTE-ACS and discharge during the early stages of presentation, we have yet to observe a significant reduction in overnight admissions in the low-risk group.

Whilst there is a clear trend in uptake of the protocol following its implementation, it is evident that it is still not being used universally across the services. This may reflect hesitancy amongst clinicians to discharge patients soon after presentation, without a significant period of monitoring. It is of paramount importance to involve all staff in understanding the rationale for change, optimising operation procedures to ensure rapid turn-around times for sequential blood draws and to streamline a rapid assessment process; in order to reap the benefits of an earlier rule-out.

This study looks predominantly at the rule-in/rule-out power of the ESC algorithm at 0 hour, based on a hs-cTnT measurement at presentation. Whilst we have been able to retrospectively quantify the risk classification of patients based on ΔTnT, the translation of ΔTnT values into prospective clinical practice needs further evaluation. Although the ESC recommends a 0 h hs-cTnT ≥52 ng/l as a rule-in threshold, our algorithm defines rule in as >50 ng/l for ease of clinical implementation.

Chest pain is clearly the typical presentation of NSTE-ACS. However, the ESC guideline appreciates that ACS can present atypically as ‘epigastric pain, indigestion-like symptoms and isolated dyspnea’.^3^ The 0–1 h ESC algorithm suggests progression to biomarker risk stratification in the patient with ‘suspected Non-ST elevation Myocardial Infarction’ and does not delineate that this suspicion must arise from the presence of typical chest pain. As such, presenting complaints like isolated shortness of breath and abdominal pain, that feature in Figure 1, can reasonably enter the troponin algorithm if the clinician has a high index of suspicion for ACS. Nonetheless, a limitation of this study is the underlying assumption that all patients who had a hs-cTnT measured in the ED correctly entered the diagnostic algorithm, i.e. had a clinical presentation compatible with a NSTE-ACS. Further, the ‘presenting complaint’ entered on the ED triage system is more a clerical than a medically driven assessment and captures only the main complaint, and not a complex presentation. This may explain why a significant number of patients with an initial troponin in the 5–50 ng/l group did not go on to have a repeat (as it became evident that they should not have entered the algorithm in the first place). Nonetheless, this is likely to represent the reality of a patient’s clinical pathway in ED. The ESC guideline acknowledges that deviation from the protocol is appropriate in circumstances of clinical concern, and rapid rule-out is inappropriate for patients presenting very early after the onset of chest pain. Our study does not account for these possible extenuating circumstances. Importantly, despite our clinical practice moving toward faster repeat troponin measurements, the current study of ΔTnT is based on the repeat troponin at any time within 24 h, whereas the ESC guideline is predicated on a repeat at one hour. In keeping with previously published observations,^9^ approximately 12% of initial troponin samples taken in the ED were haemolysed. These samples were excluded from analysis as they inevitably lead to deviation from the algorithm, and this study aimed to look at the routine functioning of the algorithm in clinical practice. However, it is important to acknowledge that, in the real-world setting, haemolysis is likely to affect the timings of samples. Finally, the troponin values available electronically to clinicians are rounded to the nearest integer, which may lead to some discrepancy between the true risk bracket that the patient belonged to and the risk bracket that they were ascribed to clinically in ED.

## Conclusions

A 0 hour rule-in/rule-out algorithm, modelled on the 2015 ESC guideline, can be implemented with good uptake within the first few months of introduction. Although this has failed to demonstrate reduced overnight admission in the low-risk group, the algorithm clarifies the appropriate clinical pathway for up to 53% of chest pain patients at presentation. Further studies are needed to address the implications of one-hour repeat testing in routine clinical practice.
